# Diseases of Women: A Manual of Gynecology Designed for the Use of Students and General Practitioners

**Published:** 1899-04

**Authors:** 


DISEASES OF WOMEN: A Manual of Gynecology Designed for the Use of Students and General Practitioners. By F. H. Davenport, A. B., M. D., Assistant Professor in Gynecology, Harvard Medical School; Assistant Surgeon to the Free Hospital for Women,

Boston. Third edition, revised and enlarged, with 156 illustrations. Published by Lea Bros. & Co., Philadelphia. Pages, 390. Price, cloth $1.75, net.

This work, as set forth in the preface to the first edition, is designed, not as a complete text-book, covering all gynecological subjects in a general way, but to fully set forth to students and general practitioners the commoner subjects of gynecology in detail. This design the author has faithfully carried out, so that though the scope is limited, the more common, and hence the more important, gynecological questions are thoroughly discussed in an intensely practical manner.

That the idea is a popular one is attested by the work having gone through three editions in nine years. The third edition is thoroughly up to date, and the physician who will place it in his library will find himself referring to it much more frequently than he will to more pretentious works. J.

